# Molecular Identification and Expression Analysis of Filaggrin-2, a Member of the S100 Fused-Type Protein Family

**DOI:** 10.1371/journal.pone.0005227

**Published:** 2009-04-22

**Authors:** Zhihong Wu, Britta Hansmann, Ulf Meyer-Hoffert, Regine Gläser, Jens-Michael Schröder

**Affiliations:** Department of Dermatology, University Hospital of Schleswig-Holstein, Kiel, Germany; Tufts University, United States of America

## Abstract

Genes of the S100 fused-type protein (SFTP) family are clustered within the epidermal differentiation complex and encode essential components that maintain epithelial homeostasis and barrier functions. Recent genetic studies have shown that mutations within the gene encoding the SFTP filaggrin cause ichthyosis vulgaris and are major predisposing factors for atopic dermatitis. As a vital component of healthy skin, filaggrin is also a precursor of natural moisturizing factors. Here we present the discovery of a member of this family, designated as filaggrin-2 (FLG2) that is expressed in human skin. The *FLG2* gene encodes a histidine- and glutamine-rich protein of approximately 248 kDa, which shares common structural features with other SFTP members, in particular filaggrin. We found that *FLG2* transcripts are present in skin, thymus, tonsils, stomach, testis and placenta. In cultured primary keratinocytes, *FLG2* mRNA expression displayed almost the same kinetics as that of *filaggrin* following Ca^2+^ stimulation, suggesting an important role in molecular regulation of epidermal terminal differentiation. We provide evidences that like filaggrin, FLG2 is initially expressed by upper granular cells, proteolytically processed and deposited in the stratum granulosum and stratum corneum (SC) layers of normal epidermis. Thus, FLG2 and filaggrin may have overlapping and perhaps synergistic roles in the formation of the epidermal barrier, protecting the skin from environmental insults and the escape of moisture by offering precursors of natural moisturizing factors.

## Introduction

The skin functions as an effective barrier protecting the body from potentially damaging environmental influences [1]. The surface-exposed epidermis, a self-renewing stratified squamous epithelium composed of several layers of keratinocytes, is most important for the barrier defense against these challenges [2]. Keratinocytes in the outmost stratum corneum (SC) of the epidermis are sloughed off and replaced by newly differentiated cells originating from epidermal stem cells located in the basal layer [3], [4]. The cornified envelope (CE), a prominent feature of the SC, is a rigid and insoluble protein structure, which determines essential properties of the barrier function [5], [6].

Over the last two decades much effort has been made to uncover the molecular bases underlying the CE barrier function [6]. In humans many genes, involved in formation of the CE or in regulation of keratinocyte terminal differentiation, are clustered tightly within the epidermal differentiation complex (EDC), a 1.9-Mb locus on human chromosome 1q21 [7], [8]. With the exception of two single genes encoding involucrin and loricrin [9], [10], they are organized into four gene clusters: small proline-rich region genes, encoding proteins that are cross-linked to the CE proteins [11], [12]; late cornified envelope genes, encoding a group of proteins that have distinct functions [13]; S100 genes, encoding a large family of EF-hand calcium-binding proteins involved in the regulation of various cellular processes including cell growth and cell cycle regulation, differentiation, transcription and motility [14], [15]; and S100 fused-type protein (SFTP) genes (see below).

A total of five genes have been identified within the SFTP gene cluster in humans and mice, including *filaggrin* (*FLG*) [16], [17], *trichohyalin* (*TCHH*) [18], *hornerin* (*HRNR*) [19]–[21], *repetin* (*RPTN*) [22], [23] and *cornulin* (*CRNN*) [24], [25]. SFTP genes consist of three exons, a tiny untranslated exon 1, a small exon 2 containing the translation start site and encoding a 46-residue S100 domain, and a large exon 3 encoding an EF-hand domain and internal tandem repeats, which are characteristic of CE precursor proteins. The best characterized SFTP gene is the *FLG* gene encoding profilaggrin, a precursor protein generating mature filaggrin units. The liberated filaggrin is an essential component of the SC that participates in the aggregation of keratin filaments into bundles and promote the flattened shape of dead-cell remnants [6], [16], [26], while the amino-terminal domain of filaggrin enters the nucleus, where it may have a role in regulating terminal differentiation [27]. In the other hand, filaggrin is eventually degraded into free amino acids that carry out various functions in the upper cornified cells including water retention by forming part of the Natural Moisturising Factors (NMF) of the SC. Trichohyalin, another member of the SFTP family, functions in specialized epithelial tissues by contributing to greater mechanical strength [28], [29]. In hair follicles, trichohyalin is cross-linked to either itself or other CE proteins by transglutaminases 3 (TGase3) as the main constituent of the inner root sheath (IRS) and the medulla of the hair shaft [29]–[32], whereas filaggrin is proposed to be cross-linked by TGase1 [6], [33] and is specially localized in the granular and cornified cells that surround hair follicles [34], [35]. Recently we have identified hornerin as components of the SC and the outer root sheath (ORS) of normal human hair follicles [21]. These findings suggest different SFTP members play distinct roles in skin barrier function.

It has been discovered that common loss-of-function mutations within the FLG gene cause ichthyosis vulgaris, one of the most common heritable disorders of cornification, and represent major risk factors for atopic dermatitis (AD) and secondary allergic diseases [36]–[38]. These studies underline the key role for impaired skin barrier function in the development of AD. However, the complexity of the AD genetics cannot be completely explained through studies on FLG pathology, suggesting additional modifying genes may also contribute to the phenotypic spectrum of AD [39]. Although the SFTP gene cluster has long been known, the gene content of this cluster remains unclear.

A recent study reported the presence of partial mRNA encoding filaggrin-2 (FLG2), a novel member of the SFTP gene family, in human keratinocytes and described its gene structure and tissue expression [40]. Here, we report complete identification and detailed expression analysis of this new gene. We found FLG2 expression in several tissues including healthy human skin obtained from different body sites and an upregulation of *FLG2* mRNA upon Ca^2+^ stimulation in cultured keratinocytes. We could demonstrate that the filaggrin-2 protein is proteolytically processed and deposited in the epidermis and is localized in the granular and cornified layers of human skin.

## Results

### Identification of the human filaggrin-2 gene within the SFTP genes cluster

**Figure 1:**
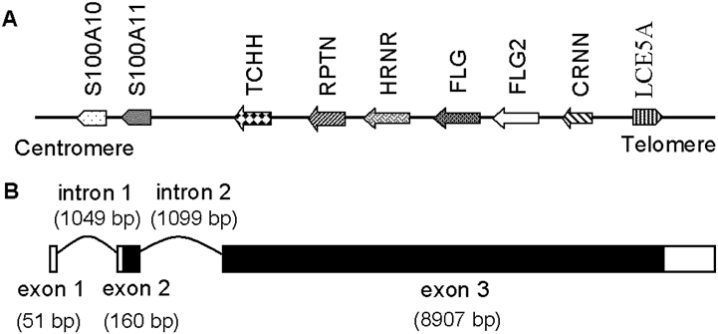
Molecular identification of the *FLG2* gene. (A) Schematic physical map of human SFTP genes locus (1q21.3). Genes are ordered from centromere to telomere. TCHH, trichohyalin; RPTN, repetin; HRNR, hornerin; FLG, filaggrin; FLG2, filaggrin-2; CRNN, cornulin; LCE5A, late cornified envelope 5A. (B) Schematic diagram of the *FLG2* gene, based on a composite cDNA isolated from foreskin-derived keratinocytes identified by overlapping RT-PCR and RACE-PCR. It consists of three exons and two introns. The positions of the exons (boxes) and introns (curve lines) of *FLG2* are deduced by comparing its full-length cDNA sequence with the corresponding genomic DNA. 5′- and 3′-untranslated regions and coding sequences are indicated by empty and black-filled boxes, respectively. The full-length nucleotide sequence has been submitted to the Gen-Bank™ Bank with the accession number AY827490.

To search for additional S100 fused-type proteins within the SFTP genes cluster, a BLAST search with the N-terminal 82 amino-acid sequence of filaggrin was performed and identified several putative gene loci. One of these hits was located within the EDC region harbouring the SFTP gene cluster and between the two known genes *FLG* (GenBank NM_002016) and *CRNN* (GenBank NM_016190) (Fig. 1A). We then retrieved this theoretical 82 amino-acid sequence to screen for matching candidates in available databases for human peptide, cDNA, and EST sequences but failed to find any hit (data not shown), suggesting that this genomic locus represents a novel gene. In view of its gene location, we proposed that this gene may be a new member of the SFTP gene family. To investigate this possibility, a theoretical partial cDNA sequence was generated by predicting the exonic sequence around this genomic locus with the FSPLICE program. This method allowed us to find two potential exons encoding the related full-length amino-acid sequence of a protein.

To verify the predicted sequence and determine its full-length cDNA sequence, a strategy combining 5′/3′RACE and short-range overlapping PCR was used. The 5′RACE and the nested PCR with reverse-transcribed RNAs from human foreskin-derived primary keratinocytes resulted in only one product. Mapping of this 5′-extended sequence determined the first exon (51 bp), the second exon (160 bp) and a part of the third exon (Fig. 1B). The 3′-extended sequence was determined by 3′RACE, which includes a polyadenylation signal site (AAUAAA) situated eleven nucleotides from its 3′ extremity. After further verification of the full-length cDNA sequence (9147 bp) with short-range overlapping PCRs, it was registered as *ifapsoriasin* with the gene symbol *IFPS* under the GenBank accession number AY827490. Later on, an approved gene name *filaggrin-2* (gene symbol *FLG2*) was suggested by the HUGO/GDB Nomenclature Committee. Comparison between the cDNA and genomic sequences of human *FLG2* revealed the presence of 3 exons and 2 introns (Fig. 1B). All the splice donor and acceptor sites conform to the GT/AG rule.

### FLG2 encodes a S100 fused-type protein

**Figure 2:**
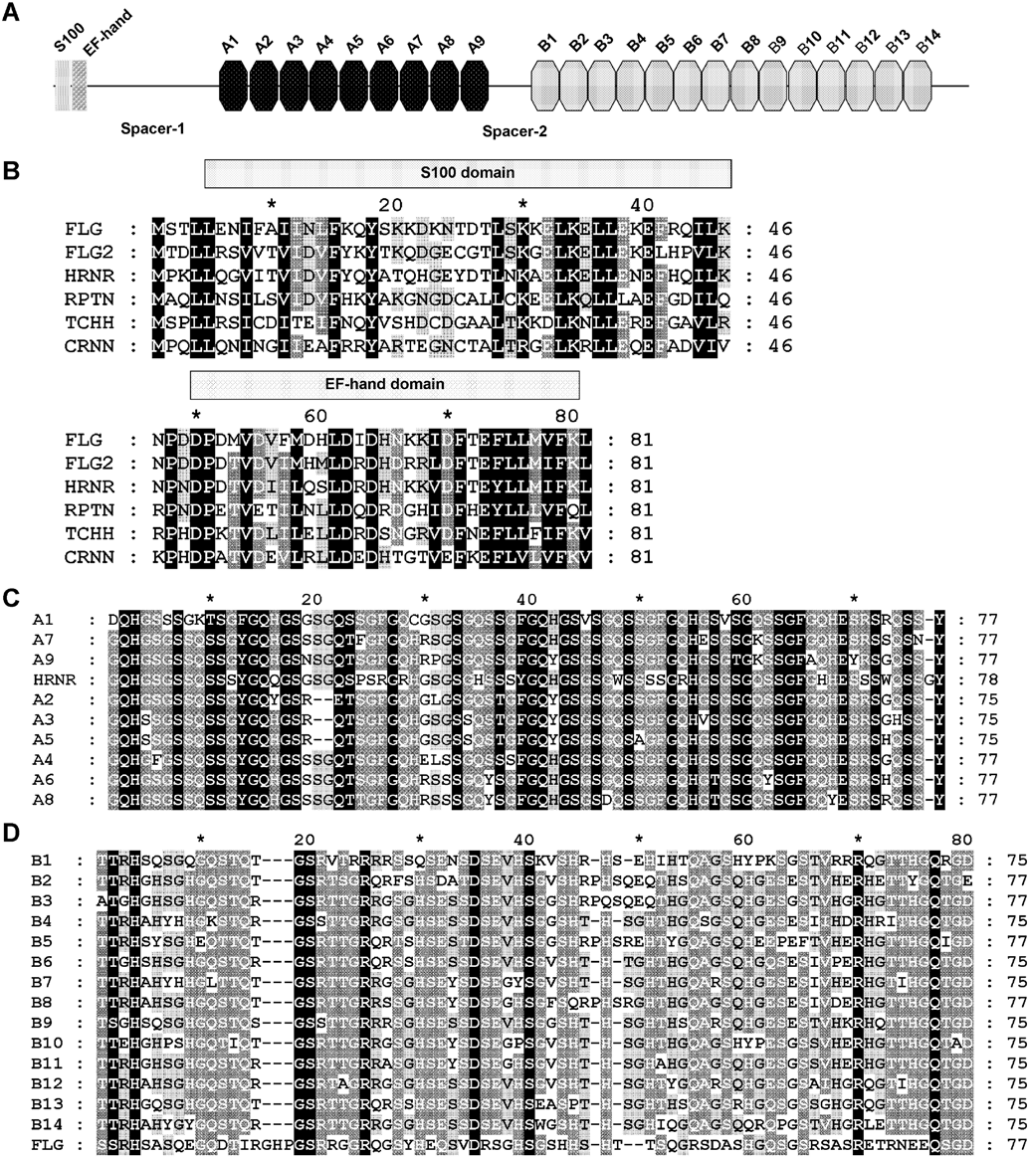
Structural characteristics of the filaggrin-2 protein. (A) A schematic diagram of human filaggrin-2. The N-terminal S100 and EF-hand domains were detected with the SMART algorithm. Repeat domains (A1 to 9 and B1 to14) were first detected by the RADAR program and then refined manually. (B) Multiple sequence alignment of N-terminal S100 domains from all known SFTP proteins. The alignment was generated by using M-COFFEE and displayed by using GeneDoc. Identical residues are boxed in black while gray boxes mark partially conserved residues. The darker the shading, the more the amino acids are conserved among SFTP members. The positions of the S100 and EF-hand regions are indicated above the alignment. FLG, filaggrin; FLG2, filaggrin-2; HRNR, hornerin; RPTN, repetin; TCHH, trichohyalin; CRNN, cornulin. (C–D) Multiple sequence alignment of A-type (C) and B-type repeats (D) of FLG2, respectively. The alignment was generated by using M-COFFEE and displayed by using GeneDoc. Identical residues are boxed in black while gray boxes mark partially conserved residues. The darker the shading, the more the amino acids are conserved among repeat domains. Here, HRNR represents hornerin (GeneBank Accession No. GI:57864582) residues 1807–1884; FLG represents profilaggrin (GeneBank Accession No. GI:60097902) residues 3745–3821.

The *FLG2* gene contains an open reading frame of 7176 nucleotides, encoding a protein of 2391 amino acids. By using SMART to explore domain architectures, this deduced protein sequence harboured an S100/ICaBP type calcium binding domain (residues 4–46) [41] and an EF-hand domain (residues 53–81) at its amino terminus. Analysis of the repeat contents using the computer program RADAR, followed by manual adjustment, revealed that this protein contains a large central repetitive region consisting of two types of multiple tandem repeats (Fig. 2A). Amino acid sequence comparison of all currently known human SFTPs, including profilaggrin, trichohyalin, repetin, cornulin, hornerin and filaggrin-2, indicated that they are highly conservative in inferring homology of the N-terminal 81-residue region containing the S100 and EF-hand domains (Fig. 2B).

**Figure 3:**
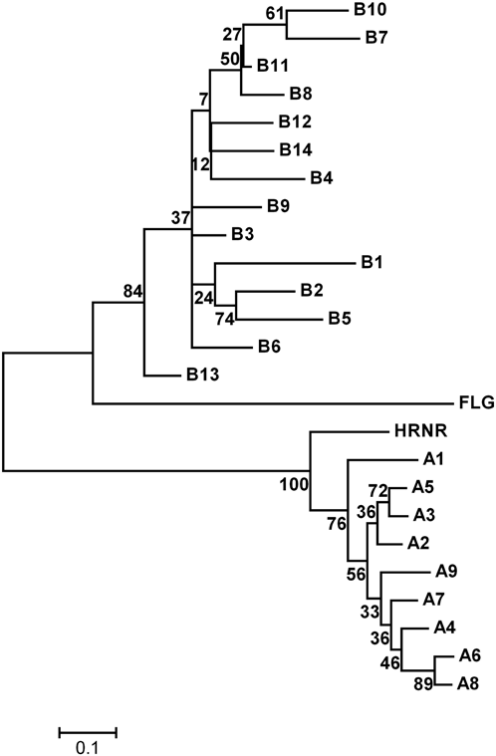
Unrooted tree of filaggrin-2 repeat domains together with typical domains of hornerin and profilaggrin. This neighbour-joining cladogram tree [68], depicting relationships among A- and B-type repeats, is constructed based on the alignment. The bootstrap consensus tree is inferred from 1000 replicates [69]. Branches corresponding to partitions reproduced in less than 50% bootstrap replicates are collapsed. The tree is drawn to scale, with branch lengths in the same units as those of the evolutionary distances used to infer the phylogenetic tree. All positions containing gaps and missing data were eliminated from the dataset (complete deletion option). There were a total of 71 positions in the final dataset. Phylogenetic analyses were conducted in MEGA4 [66]. Numbers in nodes correspond to bootstrap support values indicated as percentages.

In particular, the filaggrin-2 protein is characterized by two arrays of ordered repetitive structures separated by a spacer sequence (residues 1154–1222) (Fig. 2A). The A-type repetitive domain region is located between residues 467 and 1153, the middle part of filaggrin-2. It is comprised of nine conserved repeats, each having a size of 75∼77 amino acids (A1 to A9) characterized with four regularly spaced phenylalanine residues twelve amino acids apart (Fig. 2C). The B-type repetitive domain region is located between residues 1246 and 2303 and just in front of the C-terminal 88-residue domain. It is comprised of fourteen conserved repeats of 75∼77 amino acids (B1 to B14), but these repeats do not have any regularly spaced phenylalanine (Fig. 2D). By using these tandem repeats as queries in BLAST homolog searches against the human protein database, the most positive hit homologous to each A-type repeat was from repetitive domains of hornerin (50–77% identity) whereas that homologous to each B-type repeat was from filaggrin units (28–39% identity) (data not shown). Multiple alignments of these repeat domains together with one representative peptide of their corresponding homolog, respectively, further addressed their homology relationships (Fig. 2C and D). In addition, a neighbour-joining tree was constructed based on alignment of all FLG2 repeats together with their representative homologous repeat units of filaggrin and hornerin, demonstrating different evolutionary histories for the A-type and B-type repeats (Fig. 3).

Analysis of the overall amino acid composition of both the A-type and B-type domain revealed that they are particularly rich in two hydroxyl amino acids serine and threonine: from 32.61% in A-type repeats to 32.23% in B-type repeats. In addition, the A-type and B-type repeats have high glycine contents of 28.82% and 17.39%, respectively; the A-type repeats have high glutamine contents of 14.99%, whereas the B-type repeats have less glutamine (8.41%) but instead more basic amino acids histidine (14.93%) and arginine (8.32%). Most repeats except A1, A7, A9, B1 or B4 lack any lysine donor residue, yet rich with glutamine acceptor residues, raising the possibility that these repeat units may act as a glutamine acceptor site for the formation of transglutaminase-catalyzed N^ε^(γ-glutamyl)-lysyl isopeptide bonds [42].

### Expression of FLG2 mRNA

**Figure 4:**
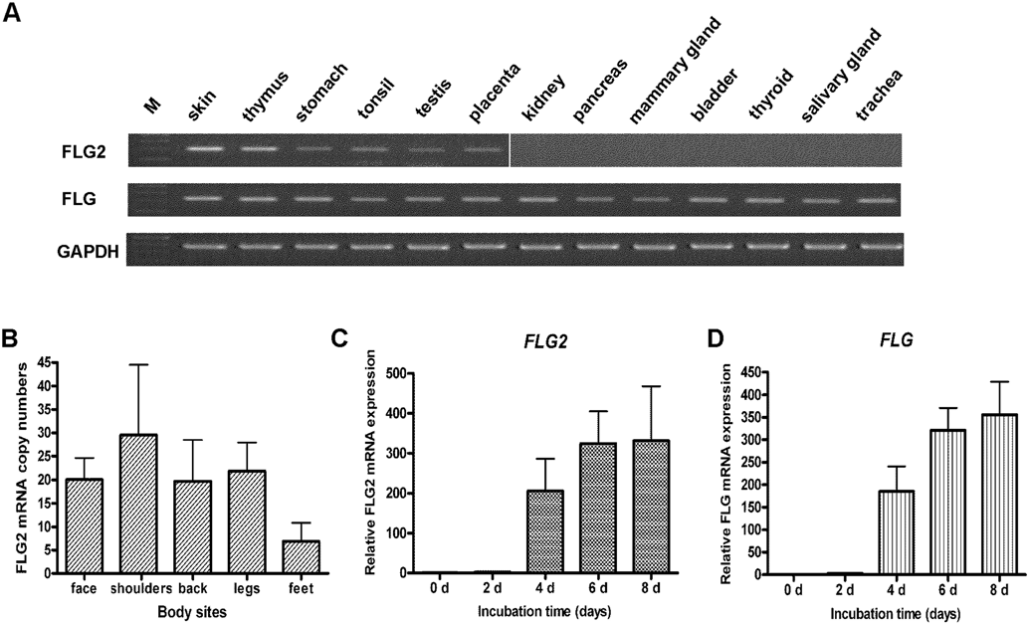
Expression analyses of *FLG2* transcripts. (A) Expression profile of *FLG2* and *FLG* mRNA. Fragments were obtained after RT-PCR amplification on multiple human tissue cDNAs with primers specific to human *FLG2*, *FLG and GAPDH*. Lanes are labelled according to the template tissue. M, DNA molecular weight marker. (B) *FLG2* mRNA is expressed at various localizations of human skin. The mRNA expression level of *FLG2* was measured by quantitative real-time PCR in human skin samples. Data were obtained from three independent experiments with different samples of skin and are indicated as absolute mRNA copy number per 10 ng of total RNA and as the mean+/−SD. Feet, the sole of the foot. (C–D) Comparative analyses of *FLG2* (C) and *FLG* (D) expression in cultured primary keratinocytes. Quantitative real-time PCR was conducted on total RNA samples collected from keratinocytes treated with 1.0 mM CaCl_2_ for the indicated times. Bar graphs represent the relative mRNA expression of either *FLG2* or *FLG* against *GAPDH*. Data are obtained from three independent trials with different sources of keratinocytes and are indicated as the mean+/−SD.

To determine the expression profile of *FLG2*, we first compared its expression with that of the *FLG* gene by using reverse transcriptase PCR (RT-PCR) analyses. As shown in Fig. 4A, both transcripts were detected in skin, thymus, stomach, tonsils, testis and placenta, but not in heart, brain, liver, lung, bone marrow, small intestine, spleen, prostate, colon, or adrenal gland (data not shown). Whereas *FLG* transcripts were detected in kidney, pancreas, mammary gland, bladder, thyroid, salivary gland and trachea, *FLG2* transcripts were not (Fig. 4A). To further analyze *FLG2* gene expression in human healthy skin, various tissue specimens from different body sites were investigated by quantitative real-time PCR. *FLG2* was expressed in all normal skin samples tested (Fig. 4B). In addition, *FLG2* mRNA level did not show significant differences among different localizations investigated and its absolute copy number was about 6–30 copies per 10 ng of total RNA.

To better understand its ubiquitous expression in the skin, *FLG2* mRNA expression was further investigated in cultured keratinocytes derived from human foreskin. In response to Ca^2+^ stimulation, a well-known stimulus to trigger keratinocyte differentiation *in vitro* [43], [44], the relative expression level of *FLG2* transcripts was elevated ∼200-fold after four days and continuously increased during further keratinocyte differentiation (Fig. 4C). As a control to monitor the keratinocyte differentiation, the *FLG* transcript level was also observed and increased similar to that of *FLG2* transcripts (Fig. 4D). This observation coincided with the observation that *FLG2* is only expressed in the uppermost epidermal layers of healthy skin (see below), supporting the idea that terminally differentiating keratinocytes represent the major cellular source of FLG2 in the skin.

### The filaggrin-2 protein is localized to the granular and horny layers of the epidermis

**Figure 5:**
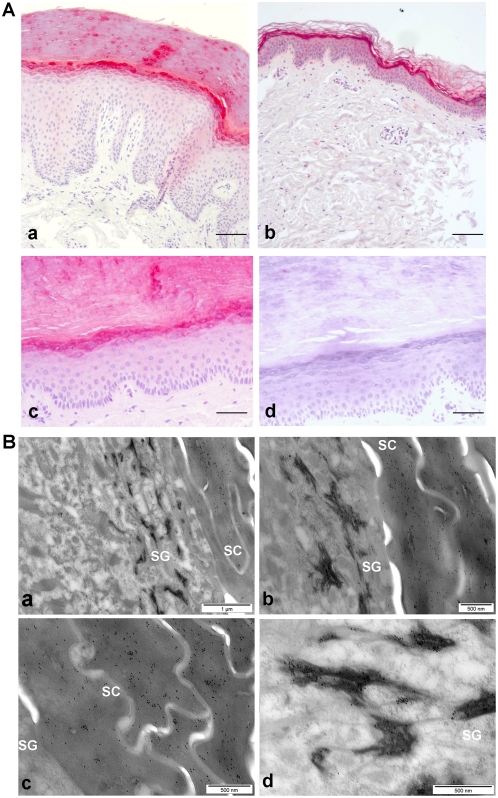
Localization of filaggrin-2 in human epidermis. (A) Immunohistochemical analyses of FLG2 in human skin sections. Healthy skin sections from palmar (panel a; scale bar, 60 µm) and plantar (panel c; scale bar, 120 µm) sites as well as the upper leg region (panel b; scale bar, 60 µm) were stained with anti-FLG2 antibody. Specificity of antibody was confirmed by blocking the antibody with the antigen (panel d; scale bar, 60 µm). Representative experiments out of three are shown. (B) Post-embedding immunoelectron microscopy of FLG2 (10 nm gold) in healthy foreskin. FLG2 labels are present in both granular and cornified cells (panels a and b). Note that FLG2 labels are either diffusely dispersed in the cytoplasm (panel c) or grouped as keratohyalin granules (panel d). SG, stratum granulosum; SC, stratum corneum.

To evaluate the expression of the FLG2 protein, goat polyclonal antibodies against rFLG2-S were generated and used for analysis of FLG2 expression in healthy human tissues and cells. The FLG2-S peptide was used for immunization because it is located within the spacer region of FLG2 exhibiting the least sequence similarity to the other SFTP members. In immunohistochemical studies, the FLG2 immunoreactivity was detected weakly in the esophagus, tonsils and testis (Fig. S1), but strongly in the stratum granulosum (SG) and stratum corneum (SC) layers of human skin (Fig. 5A). All skin samples investigated exhibited the same staining pattern. Exemplarily, localizations of immunoreactive FLG2 in palmar (Fig. 5A-a) and plantar skin (Fig. 5A-c) as well as in the facial region (Fig. 5A-b) are shown. In scalp hair follicles, the FLG2 immunoreactivity was found to be mainly restricted within the granular and cornified cells surrounding the infundibular outer root sheath (ORS) though weak immunoreactivity was also present in central and proximal ORS (Fig. S1).

**Figure 6:**
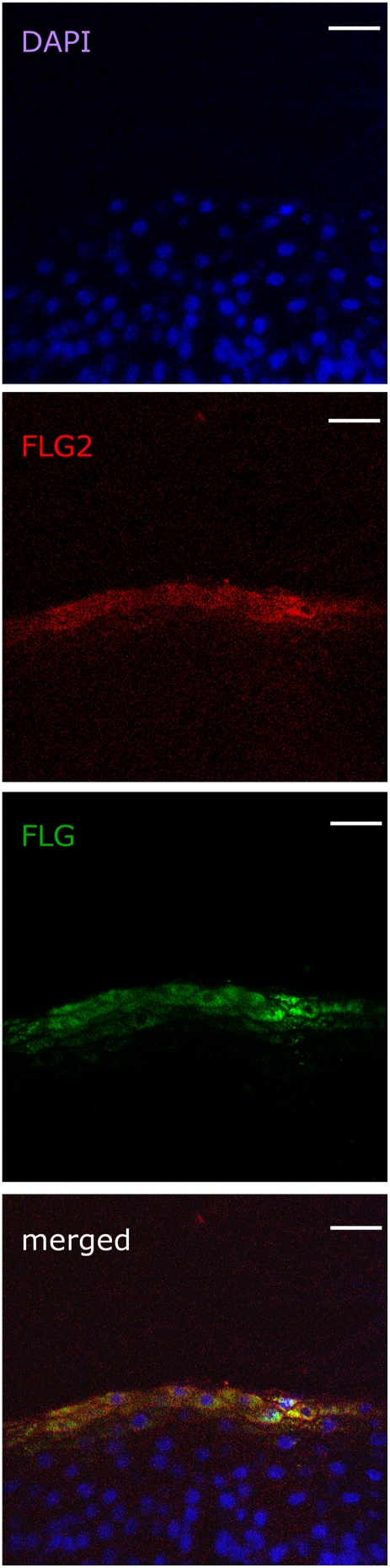
Immunofluorescence localization of FLG2 and FLG in human skin. Skin sections were stained with a mixture of anti-FLG2 and anti-FLG antibody. The images show FLG2-Alexa-Fluor as red while FLG-FITC as green. Nuclear staining was done using DAPI. Comparative localization of FLG2-Alexa-Fluor and FLG-FITC is shown in the merged image. Scale bars, 30 µm.

Sub-cellular localisation of FLG2 was further analyzed by immunoelectron microscopy. By using the LR-White resin-embedded samples of prefixed skin biopsy specimen from the knee, FLG2 labelling was observed in both, the granular and cornified cell layers (Fig. 5B-a and -b). FLG2 labels were dispersed diffusely throughout the cytoplasm in the SC, while in the SG, FLG2 was localized to irregularly-shaped keratohyalin granules of various sizes (Fig. 5B-c and -d). These data suggested that the localization of FLG2 is similar to that of filaggrin, as previously reported [45]. Therefore, the spatial localisation of FLG2 in comparison with that of filaggrin was further analysed by immunofluorescent staining and laser scanning confocal microscopy. As shown in Fig. 6, both FLG2 and FLG were mainly expressed in the granular layers of human skin. Although the merged image showed that they exhibit similar spatial distribution, only partial co-localization was observed.

**Figure 7:**
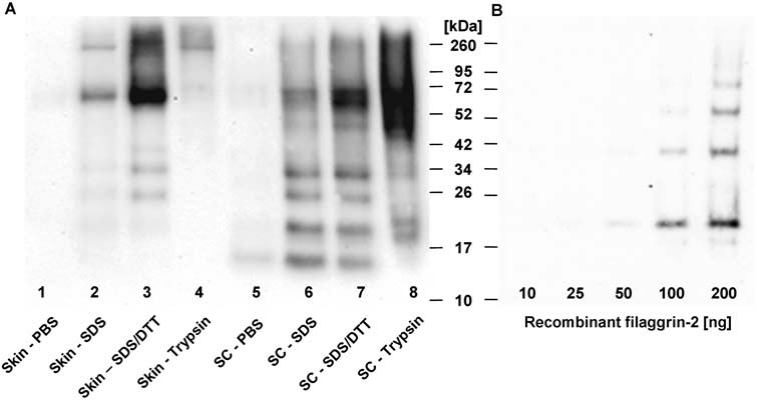
Western blot analysis of filaggrin-2 in human skin. (A) Total proteins were individually extracted from SC and abdominal epidermis, separated on an SDS-10% PAGE and blotted on a nitrocellulose membrane. The FLG2 protein was detected by using purified goat anti-FLG2 polyclonal antibody and HRP-conjugated mouse anti-goat IgG as the second antibody. Proteins were extracted sequentially from abdominal epidermis with PBS buffer (lane 1), SDS buffer (62.5 mM Tris, 10% glycerol, 5% SDS) (lane 2), and SDS/DTT buffer (SDS buffer plus 10 mM DTT) (lane 3), and from trypsin-digested skin sample (lane 4); alternatively, proteins were sequentially extracted from SC with PBS buffer (lane 5), SDS buffer (lane 6) and SDS/DTT buffer (lane 7), and from trypsin-digested SC sample (lane 8). In all other lanes, 5 µl of protein extracts were loaded. The approximate size of the protein was determined using high-molecular-weight standards. The experiment was performed twice and a representative example is shown. (B) As a control, Western blot analysis of the purified recombinant FLG2 with a theoretical monomolecular mass of 15.66 kDa was performed under the same assay conditions. Different amounts of protein were loaded as indicated. The ladder-like bands appeared with higher amounts of protein.

To get a more deep insight into its biological function, we performed Western blot analyses to examine FLG2 expression and protein processing in the skin (Fig. 7A). Using detergent-free conditions, we observed very faint staining in both the epidermal extract (∼65 kDa) and the SC extract (∼65 kDa and ∼15 kDa) (lane 1 and 5). When extraction was performed in the presence of SDS, the epidermal extract exhibited two strong bands, one corresponding to the expected molecular mass of full-length FLG2 (∼248 kDa) and another corresponding to a fragment at ∼65 kDa (lane 2). Subsequent treatment of the extract with DTT-containing buffer resulted in extraction of additional FLG2 bands corresponding to 40, 30, 25, and 20 kDa size, respectively (lane 3). In contrast, SC extracts exhibited different band patterns with stronger small-size FLG2 bands, especially a 15-kDa size band, but without a clear band at the size of full length FLG2 (lane 6 and 7). Interestingly, extracts obtained from further treatment of the SC sediment with trypsin did not abolish any FLG2-immunostaining but resulted in broad intense bands between 45 and 72 kDa (lane 8).

### Filaggrin-2 expression in psoriasis vulgaris and atopic dermatitis

**Figure 8:**
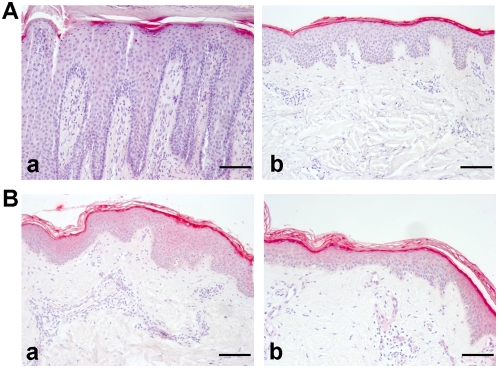
Immunohistochemical analyses of filaggrin-2 expression in skin lesions of psoriasis and atopic dermatitis. Skin biopsies from affected and non-affected sites (upper back) on patients with psoriasis or atopic dermatitis (AD) were stained with anti-FLG2 antibody. A) panel a, lesional psoriatic skin; panel b, non-lesional psoriatic skin; B) panel a, lesional AD skin; panel b, non-lesional AD skin. Representative results of three different patients of each group are shown. Scale bars, 60 µm.

To characterize FLG2 expression in inflammatory cutaneous diseases, lesional and non-lesional skin of patients suffering from psoriasis and atopic dermatitis were investigated. Psoriatic lesions (n = 3) exhibited decreased FLG2 immunoreactivity, whereas the non-lesional skin of the same patients exhibited a similar staining pattern as that of healthy individuals (Fig. 8A-a and -b). In contrast, there were no obvious staining differences observed between the lesional skin of AD patients and the non-lesional skin of the same patients and healthy individuals (n = 3; Fig. 8B-a and -b).

## Discussion

In the current study we have identified and characterized the *FLG2* gene (GenBank accession number AY827490) as a member of the SFTP gene family. A recent paper described this gene based on the registered AY827490 gene and publicly available DNA data [40]. While supporting keratinocyte expression of *FLG2* as the major cellular source [40], our data are inconsistent with their conclusion that *FLG2* expression is restricted to the epidermis. Our data show that in addition to skin, *FLG2* mRNA is also present in thymus, stomach, tonsils, testis and placenta (Fig. 4). This discrepancy may be the result of the inherent technique difficulties of RT-PCR analysis, which we have previously addressed [21]. Indeed, in consideration of high sequence similarities between each other among the 5′-end regions of all the SFTP gene members, it seems that the design of specific primers emerges as a key barrier to successfully distinguish various SFTP members. In our study, we have developed two specific primer pairs that can uniquely amplify the *FLG2* gene with the Primer Unique program.

In contrast to the proposed classification of the FLG2 repeats [33], we suggest that FLG2 can generate two kinds of repeat units with homology to filaggrin and hornerin, respectively (Fig. 2). There are nine A-type internal repeats, which display high identity to hornerin, and fourteen B-type internal repeats, which resemble those of filaggrin. The phylogenetic tree constructed based on their amino acid sequences supports the above idea (Fig. 3). Furthermore, both repeat domains of FLG2 contain a high proportion of histidine and glutamine residues, the source of the natural moisturizing factors urocanic acid and pyrrolidone carboxylic acid, suggesting FLG2 may contribute to maintaining SC hydration as proposed for filaggrin [46].

Similar to that of the *FLG* gene [16], [47], the final huge exon of the *FLG2* gene contains highly GC-rich repeats with similar nucleotide sequences. Such properties may cause technical problems for PCR-based amplification and subsequent sequencing, as was also reported for analyzing the *plectin* gene [48] and the *FLG* gene [49]. The reason is that GC-rich templates frequently generate local secondary structures leading to an inefficient PCR amplification [50]. Therefore, it is a technical challenge to efficiently clone and sequence the full-length cDNA of this kind of genes. In this study, a comprehensive strategy has been developed by combining bioinformatic analysis, RACE and short-range overlapping PCR to overcome PCR problems and subsequent sequencing steps to yield the full-length cDNA sequence of the *FLG2* gene. This strategy seems to be the best way to analyze a gene like *FLG2* with highly similar sequences and GC-rich repeats at DNA level. More importantly, the primer pairs used for successful determination of *FLG2* cDNA (Table 1) should be crucial for detailed functional analyses of the *FLG2* gene in the future. Indeed, we have failed to clone the length cDNA sequence of FLG2 by long-distance RT-PCR (data not shown).

**Table 1:** Primers used for various applications as described in the text.

Name	Sequence (5′-3′)	Location
5′-RACE-PCR:		
FLG2-R1	TGGTGTCGGTGACCACGCCTATGCTTC	400/374
FLG2-R2	GCTGAGGACCTTGTTGCAGGCCATAGTCA	343/316
3′-RACE-PCR:		
FLG2-F1	GATGTCATCATGCATATGCTGGATCGAG	237/264
FLG2-F2	AGGCTTGAGTCAGTCCTCTGGGTTCG	1376/1401
FLG2-F3	TGGGCCAGGGTGAATCTCAACAAGTAGAG	3727/3755
FLG2-F4	GGTCAACAGACAGCACTGCAAACAAGCAAC	7207/7236
FLG2-F5	TGGATATAACACAGCCAGAAGGGAGGGATG	7460/7489
FLG2-F6	GTTCGAGACCAGCCTGGCCAACATA	8490/8514
FLG2-F7	TCAGGAGGCTGAGGCAGGAGAATTG	8587/8611
RT-PCR and Real-time PCR:		
FLG2-rtF	ACCAGGGTTCACTTAAACTTGCA	47/69
FLG2-rtR	ATGACATCCACTGTGTCTGGATC	244/222
Short-range overlapping PCR:		
FLG2-R2672	TCCCGAACTTGACCCATGTTGACC	2672/2649
FLG2-F2484	TCCACTGGCTTTGGCCAATATGGA	2484/2507
FLG2-R2954	GCCAGAGGATTGACCTGAGCCTGA	2954/2931
FLG2-F3234	CATGGGACTGGTTCAGGACAATCCTC	3234/3260
FLG2-R4433	TTGAGATCCGGCTTGGCCATGAGT	4433/4410
FLG2-F4293	GGACAGTCCACACAGAGAGGGTCCA	4293/4217
FLG2-R5665	TGTCCATGACCAGATTGAGAATGTCCA	5665/5639
FLG2-F5501	TGAAGGGCACTCAGGGTTCTCACA	5501/5524
FLG2-R6838	TGGCCAGATCCCCTTCTTCCAGTT	6838/6815
Plasmid Constructions:		
Sumo-IFPS-2-F	GCTTCAGGGTCAAAGAAGCA	360/379
Sumo-IFPS-2-R	TCATTTCCTTTCCCAACTGTTTG	778/759
PET-IFPS-2-F	ACTGAGATCTGGGTACCGACGACGACGACAAGGCTTCAGGGTCAAAGAAGCA	360/379
PET-IFPS-2-R	ATTTGCGGCCGCTCATTTCCTTTCCCAACTGTTTGAT	778/759

In the epidermis and hair follicles, the localization of FLG2 is similar to that of filaggrin but is different from that of hornerin or trichohyalin [21], [45]. FLG2 immunoreactivity is present as FLG2 granules in granular cells but is diffusely distributed in the cytoplasm of cornified cells (Fig. 5). However, FLG2 and filaggrin did not show complete colocalization (Fig. 6). These data suggest that FLG2 and filaggrin might have not any direct intermolecular cross-linking between each other but may play a similar role in skin biology. Profilaggrin is an insoluble, highly phosphorylated protein, which consists of multiple FLG units joined by linker peptides [51], [52]. During terminal differentiation this protein is dephosphorylated and processed by several endoproteases to generate two major products: FLG and the N-terminal peptide [27]. FLG mainly functions to aggregate keratin filaments leading to keratinocyte compaction and formation of the SC [6] by conjugation of glutamine-rich linkage proteins with lysine-rich proteins like P-cystatin, a known cross-linking partner of filaggrin in the SC [53]. A similar mechanism can be suggested for FLG2 repeats to aggregate lysine-rich proteins because these repeats almost lack cysteine or lysine residues but contain high glutamine contents, implicating that they may act as amino acceptors in transglutaminase-catalyzed reactions to generate glutaminyl-lysine cross-links with neighbouring proteins [54]. The cross-linking possibility of FLG2 is supported by the detection of natural FLG2 proteins under reducing conditions (Fig. 7). The absence of a clear full-length FLG2 band in the SC extract suggests that FLG2 is processed within the stratum granulosum. However, because of the existence of different FLG2 intermediates in the SC, further protein processing can be expected to happen in order to completely generate mature FLG2 repeat domains. This observation is consistent with data from profilaggrin, where an N-terminal peptide of 32 kDa is released after proteolytic processing [55], suggesting the processing of the N-terminal domain of FLG2 could be similar to that of filaggrin.

Common loss-of-function mutations within the *FLG* gene causing ichthyosis vulgaris, filaggrin units represent major risk factors for atopic dermatitis (AD) and secondary allergic diseases [36]–[38]. In the investigation of FLG2 immunoreactivity in skin biopsies from patients with AD and psoriasis vulgaris, two of the most common inflammatory skin diseases, we have observed decreased expression of FLG2 only in the lesional but not in uninvolved skin of psoriasis patients. Because FLG2 expression strongly depends on terminal differentiation of keratinocytes, the decreased expression of FLG2 might be caused by the altered differentiation in the psoriatic plaques. Indeed, reduced expression of FLG has also been reported for psoriasis lesions [56], though *FLG* null alleles are not associated with psoriasis [57]. In contrast, it has been reported that hornerin, another member of the SFTP family, is strongly expressed in psoriatic lesions [20]. In AD patients, no obvious differences have been observed for FLG2 expression in lesional and nonlesional skin. Since the number of patients investigated was limited (n = 3), it might be still of interest to investigate the association of the FLG2 expression and AD in larger cohorts.

Taken together, these findings identify FLG2 as a novel member of the SFTP family present in the granular and horny layers of human epidermis, which might play a role in skin barrier networks. Future work will be necessary to determine the processing of FLG2 repeat domain units and their biological function.

## Materials and Methods

All experiments were performed according to the Declaration of Helsinki protocols and under protocols approved by the Ethics Committee at the Medical Faculty of the Christians Albrechts University, Kiel, Germany (A104/06). Normal skin specimens were taken from routine clinical work at the Department of Dermatology, UKSH Kiel, and represent tumour-free margins of benign melanocytic tumours surgically removed from patients. All specimens were obtained from patients who had signed informed consent forms. In addition, skin samples were obtained from untreated patients with AD and psoriasis vulgaris in local anaesthesia. Other tissues were derived from normal individuals posthumously during autopsy after cardiac arrest. All primers used for various applications as described in the text were designed with the Primer3 server [58] and ordered from Sigma–Aldrich (Taufkirchen, Germany) (Table 1). Restriction endonucleases were from New England Biolabs (Frankfurt, Germany). Dulbecco's PBS was obtained from PAA Laboratories GmbH (Cölbe, Germany). Acetonitril, methanol and H_2_O (for HPLC) were obtained from Promochem (Wesel, Germany). All other chemicals were ordered from Sigma–Aldrich (Taufkirchen, Germany), if not otherwise indicated.

### Culture and stimulation of keratinocytes

Foreskin-derived primary keratinocytes were prepared from neonatal foreskin after surgery following established methods [59] and were cultured in Epilife medium in 75-cm^2^ flasks (BD Biosciences, Heidelberg, Germany) in a humidified atmosphere with 5% CO_2_. For stimulation and RNA isolation, cells were grown in 12-well tissue culture plates (4 cm^2^/well; BD Biosciences) and were used after the second passage at a confluence of 70–80%. Stimulation was performed for the indicated time with 1.0 mM freshly prepared CaCl_2_.

### mRNA expression analyses

Total RNA was isolated from human skin samples or cultured foreskin-derived keratinocytes using the RNeasy Mini kit (Qiagen, Hilden, Germany). All other RNAs were obtained from BD Bioscience Clontech (Heidelberg, Germany). A total of 2 µg of total RNA was reverse transcribed with an oligo(dT)_18_ primer and Superscript II RNaseH^−^ reverse transcriptase (Invitrogen, Hamburg, Germany). The reaction mixture was diluted 10-fold with H_2_O for further PCR experiments. To design primer pairs to uniquely amplify the FLG2 gene, all the 5′-end 500-bp nucleotide sequences of the SFTP gene family were retrieved from GenBank and then used for primer designing by the Primo Unique program (http://www.changbioscience.com/primo/primou.html).

Qualitative RT-PCR assay was performed with Advantage 2 Polymerase Mix (BD Bioscience Clontech, Heidelberg, Germany). One pair of gene-specific primers FLG2-rtF and FLG2-rtR (Table 1) were designed to overlap intron 2 of the *FLG2* gene and amplify the 198-bp cDNA target. The thermal cycling conditions were as follows: firstly with 5 cycles at 95°C for 20 s, 68°C for 30 s; secondly 5 cycles at 95°C for 20 s, 66°C for 30 s; then 30 cycles at 95°C for 20 s, 63°C for 30 s, 68°C for 30 s. The PCR products were separated on a 2.0% agarose gel and then visualized by ethidium bromide staining. The specificity of the PCR products was confirmed by sequencing. As an internal control of cDNA templates, the housekeeping gene *glyceraldehyde phosphodehydrogenase* (*GAPDH*) was assessed with each cDNA in a separate PCR reaction by one pair of intron-spanning primers (forward primer: 5′-CCA GCC GAG CCA CAT CGC TC-3′, reverse primer: 5′- ATG AGC CCC AGC CTT CTC CAT-3′).

Quantitative real-time RT-PCR assay was carried out with the same primer pair as above using the SYBR® Premix Ex Taq™ Kit (Takara Bio, Heidelberg, Germany) in a fluorescence thermocycler following the instructions of the manufacturer (LightCycler, Roche Molecular Biochemicals, Hamburg, Germany). Cycling conditions were: 45 cycles at 95°C for 10 s, then 69°C for 20 s (‘touchdown’ of −1°C/cycle to 63°C). Fluorescence measurements were taken at 72°C at the end of each cycle. Amplicons were analysed by 2.0% agarose gel electrophoresis and, where necessary, purified and sequenced to confirm their identity. As an internal control to account for keratinocyte differentiation in response to Ca^2+^-stimulation, the *FLG* gene was measured with each cDNA in a separate PCR reaction using intron-spanning primers (forward primer: 5′- GGC TCC TTC AGG CTA CAT TCT A -3′, reverse primer: 5′- ATC TGG ATT CTT CAG GAT TTG C -3′). Cycling conditions were as follows: with 45 cycles at 95°C for 10 s, 69°C for 20 s (‘touchdown’ of −1°C/cycle to 63°C), 72°C for 15 s, then 76°C acquisition of signals. For calculation of the relative transcripts amplification, the housekeeping gene *GAPDH* was performed with each cDNA in a separate PCR reaction by the same pair of primers as above. For standard curve acquisition, eight serial 10-fold dilutions of linearized plasmid DNA containing the insert were prepared for each primer set, representing 30 to 3.0×10^8^ double-stranded DNA molecules quantified by use of a BioPhotometer (Eppendorf, Hamburg, Germany). For relative real-time PCR, the data from one triplicate-sample result of three independent experiments were analyzed with software (GraphPad Prism 4) and expressed as mean±SD of mRNA in question relative to that of *GAPDH*. For absolute DNA quantification, reactions were carried out with different concentrations of linearized plasmid DNA containing the insert in parallel to the samples that should be quantified. The data from three independent observations were analyzed with the software and expressed as mean±SD of mRNA copies in question relative to 10 ng of total RNA.

### *In silico* analyses

Homology search was done using the BLAT algorithm [60] as provided by the UCSC Genome Browser (http://genome.ucsc.edu/cgi-bin/hgBlat) and the BLAST algorithm [61] as provided by the Ensembl server (http://www.ensembl.org/ Multi/blastview). Subsequent sequence manipulations utilized the online BLAST 2 Sequences [62] (http://www.ncbi.nlm.nih.gov/ blast/bl2seq/bl2.html). Splice site analysis was performed using the FSPLICE program implemented at the SoftBerry server (http://www.softberry.com/berry.phtml). Protein domains were analyzed on the SMART server [63]. Internal sequence repeats were detected using the RADAR algorithm [64] and modified by manual adjustment afterwards. Multiple sequence alignments were performed using the M-coffee program [65] and Neighbor-Joining trees were constructed in MEGA4 [66]. Alignments were edited with GeneDoc (http://www.psc.edu/biomed/genedoc).

### Determination of the full-length sequence of FLG2 cDNA

Total RNA was obtained from cultured human foreskin-derived keratinocytes using TRIzol reagent (Invitrogen, Hamburg, Germany). After treatment with RNase-free DNase I (Roche Diagnostics, Mannheim, Germany) to exclude contamination with genomic DNA, 3 µg of DNA-free total RNA was then used for the first-strand cDNA synthesis for RACE (Rapid Amplification of cDNA Ends) with SMART RACE cDNA Amplification Kit (BD Bioscience Clontech, Heidelberg, Germany) according to the manufacturer's protocol. For 5′-RACE, two specific primers FLG2-R1 and FLG2-R2 (Table 1) were designed based on the nucleotide sequence of the putative exon 3 of the *FLG2* gene and used as antisense primers. For 3′-RACE, seven specific primers FLG2-F1∼7 (Table 1) were designed against the nucleotide sequence of the putative exons 2 and 3 of the *FLG2* gene and used as sense primers, among which each FLG2-F2∼7 was used for the second round PCR whereas FLG2-F1 was used for the first round PCR. RACE reactions were set up as follows: 5 µl of adapted cDNA, 0.2 µM 10×UPM primer, 0.2 µM gene-specific primer, 5 µl of 10×reaction buffer, 0.2 mM dNTP, 1 µl of BD Advantage 2 Polymerase mix, in a final volume of 50 µl. RACE PCR was performed on MJ Research Peltier Thermal cycler PTC-200 and reaction cycles were as follows: 95°C for 1 min; 5 cycles of 95°C for 20 sec, 72°C for 5 min; 5 cycles of 95°C for 20 sec, 70°C for 5 min; 25 cycles of 95°C for 20 sec, 68°C for 5 min; and a final extension of 10 min at 68°C. Then, 0.5 µl of PCR products was used as template for nested PCRs under the following conditions: 1 min at 95°C, 30 cycles of 20 sec at 95°C and 3 min at 70°C, and a final extension of 10 min at 70°C.

To characterize the full-length cDNA sequence of *FLG2*, an alternative approach was used, namely the short-range overlapping PCR strategy. Firstly, based on the newly defined 5′- and 3′-terminal sequence, the putative middle part of mRNA sequence was initially predicted by in silico analysis and then used for primer design; secondly, all subsequent PCRs used primer pairs (Table 1) designed to amplify about 0.5∼1.4 kb length of overlapping cDNA. Thirdly, to design a pair of primers, one primer maps the previously confirmed cDNA sequence while another primer maps the predicted DNA sequence to be tested. The first round 3′RACE-PCR product was used as the template for PCR using BD Advantage 2 Polymerase mix under the following conditions: 1 min at 95°C, 30 cycles of 20 sec at 95°C and 90 sec at 70°C, and a final extension of 10 min at 70°C. The amplified products were subcloned and sequenced.

### Construction of prokaryotic expression vectors for FLG2 expression in *E. coli*

Recombinant protein expression of FLG2 cDNA in *Escherichia. coli* (*E. coli*) was performed by molecular subcloning of a partial FLG2 cDNA into prokaryotic expression vectors. This partial coding region, termed FLG2-S (residues 96 to 235), was generated as a PCR fragment using *Pfu Turbo*® DNA polymerase (Stratagene, La Jolla, USA). Two expression vectors, pET-SUMO (Invitrogen) and pET-32a (Novagen, North Ryde, Australia), were used in this study. One primer pair pET-IFPS-2-F and pET-IFPS-2-R (Table 1) was used to introduce the cloning sites *Bgl*II and *Not*I sites. The PCR product was first subcloned into a pGEM-T vector (Promega, Mannheim, Germany) and then double-digested prior to being cloned into the similarly digested pET-32a vector. To be subcloned into the pET-SUMO vector, the insert was generated with the primer pair SUMO-IFPS-2-F and SUMO-IFPS-2-R. Both constructs were transformed into *E. coli* TOP10 (Invitrogen) and selected on LB agar plates containing appropriate antibiotics. Clones were sequenced to check for any mutation that might have been misincorporated during the amplification.

### Expression and purification of polyhistidine-tagged proteins

The pET-32a-derived expression construct was transformed into *E. coli* BL21*trxB*(DE3)*pLys*S cells (Novagen, North Ryde, Australia) and selected on LB agar plates containing carbenicillin (50 µg/ml), chloramphenicol (34 µg/ml) and kanamycin (15 µg/ml). The pET-SUMO-derived expression construct, on the other hand, was transformed into *E. coli* BL21(DE3)*pLys*S cells (Novagen) and selected on LB agar plates containing kanamycin (50 µg/ml) and chloramphenicol (34 µg/ml). Transformants were grown at 37°C and 200 rpm in LB medium containing appropriate antibiotics to an OD_600_ of 0.4 to 0.6. Protein expression was induced with 1 mM IPTG (isopropyl-beta-D-thio-galactopyranoside) for 3 h. After incubation, cells were harvested by centrifugation and resuspended in 1×LEW buffer (50 mM NaH_2_PO_4_, 300 mM NaCl, pH 8.0). Resuspended cells were subjected to three cycles of freeze-thawing and sonicated on ice. After centrifugation at 13,500×g for 45 min, the clarified supernatant was applied to Protino®Ni prepared columns (Macherey-Nagel, Dueren, Germany) and the polyhistidine-tagged protein was eluted with 1×elution buffer (50 mM NaH_2_PO_4_, 300 mM NaCl, 250 mM imidazole, pH 8.0). The fusion protein was further purified by reversed phase high-performance liquid chromatography (RP-HPLC) using preparative wide-pore C8 RP-HPLC with a column (SP250/10 Nucleosil 300-7 C8; Macherey-Nagel) that was previously equilibrated with 0.1% (v/v) TFA in HPLC-grade water containing 10% acetonitrile. Proteins were eluted with a gradient of increasing concentrations of acetonitrile containing 0.1% (v/v) TFA (flow rate, 3 ml/min). Fractions containing UV(215 nm)-absorbing material were collected, analyzed by ESI-QTOF-mass spectrometry (Micromass, Manchester, U.K.) and lyophilized.

### Digestion and purification of recombinant proteins

The his-tagged SUMO fusion protein purified from HPLC-RP8 was cleaved with SUMO protease 1 according to the manufacturer's protocol (LifeSensors Inc.). The digestion mixture contained 2 units of SUMO protease 1 per 100 µg of the fusion protein in a volume of 500 µl of 1×PBS buffer and was incubated for 3 h at 30°C prior to be adjusted to a pH value between 3.0 and 4.0. After a brief centrifugation, the supernatant was injected onto a Jupiter-5μ-C4-300A HPLC column (Phenomenex, Aschaffenburg, Germany) equilibrated with 0.1% TFA and 10% acetonitrile in water. Peptides were eluted with a gradient of increasing concentrations of acetonitrile containing 0.1% (v/v) TFA (flow rate, 0.5 ml/min). Fractions of each peak were collected, analyzed by ESI-QTOF-mass spectrometry and lyophilized.

### Production of Antibodies

Polyclonal antiserum was generated in goat against the amino acid sequence of human FLG2 (residues 96 to 235). From a total of 1.8 mg of protein mixture including 800 µg of the pET-32a-derived fusion protein and 1 mg of the purified recombinant protein, 1.2 mg was conjugated by the glutaraldehyde method [67] to 1.0 mg of maleimide-activated keyhole limpet hemocyanin (KLH) and subsequently mixed with 600 µg of the uncoupled protein mixture for use as immunogens. Immunization of a goat was carried out four times on days 0, 14, 28 and 35. Goats were bled 2 weeks after the last booster. The antiserum was affinity-purified by absorption against the HPLC-RP4 purified recombinant FLG2-S (rFLG2-S) that was covalently bound to HiTrap NHS-activated HP 1 ml columns (Amersham Biosciences, Freiburg, Germany). Specificity was tested by immuno-dot analyses using rFLG2-S.

### Protein extraction and Western blot analyses

Proteins were extracted from abdominal skin and pooled heel stratum corneum (SC). The subcutaneous fat tissue was separated from abdominal skin and it was minced with a scalpel blade into smaller pieces. The skin or pooled SC was ground in liquid nitrogen in the presence of washed hydrochloric acid-treated sea sand. The fine-grained powder was added with 500 µl of PBS containing Complete Protease Inhibitor Cocktail (CPIC; Roche Diagnostics, Mannheim, Germany) (buffer 1) per 100 mg of skin (or SC), vortexed thoroughly and centrifuged. Unless otherwise indicated, all centrifugation steps were carried out at 11,900×g for 10 min. After separation of the supernatant (extract 1), the sample sediment was resuspended in 500 µl of buffer 2 (5% SDS, 10% glycerol, 62.5 mM Tris-HCl containing CPIC) and centrifuged. The supernatant was collected (extract 2) and the pellet was resuspended in 500 µl of buffer 3 (buffer 2 plus 10 mM DTT and CPIC) and incubated for 1.5 h at room temperature before centrifugation. This supernatant was also collected (extract 3). Then, the sediment was washed with 100 mM NH_4_HCO_3_ to remove residual SDS and DTT and resuspended in 150 µl of 100 mM NH_4_HCO_3_ per 100 mg of original sample. This mixture sample was incubated for 5 h at 37 °C with 4 µg of activated sequencing-grade trypsin (Roche Diagnostics, Mannheim, Germany). The supernatant was pooled by centrifugation.

For Western blotting, 5 µl of total proteins were separated on an SDS-10% polyacrylamide gel (PAGE) and blotted onto a Protran® nitrocellulose transfer membrane (pore size 0.2 µm, Schleicher & Schuell BioScience, Dassel, Germany). After blocking for 1 h in 5% (w/v) nonfat powdered milk and 0.05% Tween in PBS (PBS-T), membranes were incubated at 4°C for 18 h in PBS-T containing 3% (w/v) nonfat powdered milk and the affinity-purified polyclonal anti-FLG2 antibody (1 mg/ml, 1∶4000 dilution). Membranes were washed six times for 5 min each with PBS-T and incubated for 1 h in PBS-T buffer containing 3% (w/v) nonfat powdered milk and mouse anti-goat IgG HRP conjugate (diluted 1∶20,000; Dianova, Hamburg, Germany). After six times washing prior to incubation for 5 min with chemiluminescent peroxidase substrate, the blot was visualized with a Diana III cooled CCD-camera imaging system (Raytest, Straubenhardt, Germany). All experiments were performed at least twice and a representative example was shown.

### Immunohistochemistry

Frozen 5 µm tissue sections were fixed in acetone and in 4% (v/v) paraformaldehyde before the immunoassaying. Paraffin-embedded sections were deparaffinized, rehydrated and subsequently blocked with 10% normal goat sera containing 0.1% BSA and 0.2% glycine. After brief washing in tris-buffered saline (TBS), the sections were stained with affinity-purified polyclonal goat anti-FLG2 antibody (1 mg/ml, 1∶200 dilutions) in a humid atmosphere. The sections were subsequently incubated with biotinylated anti-goat IgG (Vector, Burlingame, CA), followed by detection using the Vectastain ABC-AP® system (Vector). Haematoxylin was used for counterstaining (DAKO, Glostrup, Denmark). Specificity test of the anti-FLG2 antibody was performed by using rFLG2-S peptides to block the primary antibody. Negative controls were established by using preimmune goat sera to stain sections.

### Immunofluorescence and confocal microscopy

To show the potential spatial relationships between FLG-2 and FLG, the paraffin-embedded skin sections (5 µm) were blocked with 10% normal goat and mouse sera (Vector) in TBS containing 0.1% BSA and 0.2% glycine after standard rehydration. For double labeling, sections were treated as for single labeling and incubated with a mixture of anti-FLG2 antibody (1∶50 dilution) and a mouse monoclonal anti-filaggrin antibody (1∶200 dilution; Biologo, Kronshagen, Germany) overnight at 4°C. Subsequently, they were washed three times in PBS for 10 min and then incubated with a mixture of secondary antibodies, AlexaFluor-coupled chicken-anti-goat IgG (Invitrogen) and FITC-coupled horse-anti-mouse IgG (1∶400 each; Biologo, Kronshagen, Germany) for 1 h at room temperature. Nuclei present in the epidermal cells were counterstained with DAPI. To exclude autofluorescence secondary to the preparation of the sections, control sections were stained without primary antibodies. Slides were analyzed using a confocal laser scanning microscopy (Zeiss, LSM 510 UV, Jena, Germany).

### Immunoelectron microscopy

Fresh skin biopsy specimen from the knee were washed twice with PBS and fixed for 4 h on ice in 4% freshly depolymerized paraformaldehyde and 0.1% glutaraldehyde in 200 mM sodium cacodylate buffer (pH 7.2). The fixed tissue was washed with buffer, dehydrated in a graded series of ethanol and embedded in LR-White resin according to standard procedures. Polymerizationwas carried out at 4°C for 48 h under UV light. Ultrathin sections were prepared with an UCT ultramicrotome (Leica Microsystems, Bensheim, Germany) and placed on 50-mesh Au Grids. Ultrathin sections were washed three times for 2 min each with PBS containing 20 mM glycin and incubated in blocking buffer (0,2% fish skin gelatine, 0,2% BSA in PBS) for 10 min. The sections were incubated overnight at 4°C in anti-FLG2 antibodies, diluted 1∶100 in blocking buffer. After washing with blocking buffer (five changes, 3 min each), the sections were incubated for 1 hour at room temperature with rabbit anti-goat coupled to 10-nm gold (Biotrend, Köln, Germany), diluted 1∶50 in blocking buffer. Thereafter, the sections were washed in PBS (five changes, 3 min each), fixed in 1% glutaraldehyde (3 min) and washed in Milli-Q water (five changes, 2 min each). As a control, preimmune serum was used. The sections were counterstained for 10 min with 2% aqueous uranyl acetate (Serva, Heidelberg, Germany). Immunolabeled grids were analyzed using a transmission electron microscope CM10 (Philips, Eindhoven, The Netherlands).

## Supporting Information

Figure S1 Immunohistochemical analyses of FLG2 in tissue sections and hair follicles. Anti- FLG2 antibodies were used to stain cryosections of the kidney, esophagus, pharynx, pancreas, tonsils, testis and hair follicles. The esophagus, tonsils and testis show weak immunoreactivity while the kidney, pharynx and pancreas do not. In the hair follicle, the distal parts of the outer root sheath show the strong staining pattern, whereas in central and proximal parts the outer root sheath epithelium show weak immunoreactivity.
(3.20 MB TIF)
