# Supplementary figures and images for: Molecular Identification and Expression Analysis of Filaggrin-2, a Member of the S100 Fused-Type Protein Family
Source: PLoS One. 2009 Apr 22;4(4):e5227. doi: 10.1371/journal.pone.0005227 (PMC2668185; doi:10.1371/journal.pone.0005227)

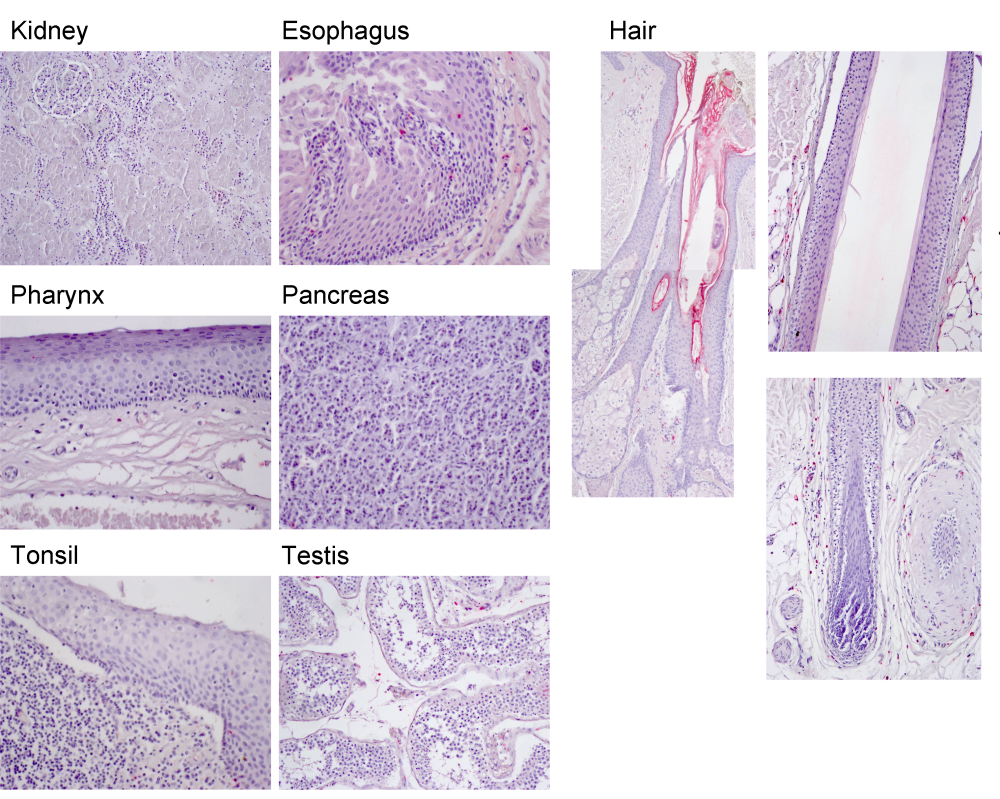

Supplement: Figure S1 — Immunohistochemical analyses of FLG2 in tissue sections and hair follicles. Anti- FLG2 antibodies were used to stain cryosections of the kidney, esophagus, pharynx, pancreas, tonsils, testis and hair follicles. The esophagus, tonsils and testis show weak immunoreactivity while the kidney, pharynx and pancreas do not. In the hair follicle, the distal parts of the outer root sheath show the strong staining pattern, whereas in central and proximal parts the outer root sheath epithelium show weak immunoreactivity. (3.20 MB TIF) [file pone.0005227.s001.tif]
